# Genotypic evidence of vector-mediated selection on *Plasmodium falciparum* in infected field-collected *Anopheles* mosquitoes

**DOI:** 10.64898/2026.10.23.759723

**Published:** 2026-10-23

**Authors:** Ailsa Craven, Ousmane Akone-Ella, Katrina A. Kelley, Theo Mouillaud, Sandrine Eveline Nsango, Sarah Perkins, Angela M. Early, Diego Ayala, Daniel E. Neafsey

## Abstract

Malaria-causing *Plasmodium* parasites are transmitted between vertebrate hosts via diverse species of *Anopheles* mosquitoes. The P47 protein in *Plasmodium falciparum* has been shown to affect transmission in various anopheline species by mediating evasion of mosquito innate immunity in an allele-specific manner. The *Pfs47* gene that encodes P47 exhibits high genetic divergence across continents, as well as high genetic diversity within many parasite populations, but the impact of polymorphisms on transmission success in different sympatric vectors has not been previously studied in a field context. Here we report parasite genotyping data from naturally infected, field-collected mosquitoes at sites harboring at least two prevalent sympatric vector species, targeting *Pfs47* in addition to the *CSP* , *AMA1* , *SERA2* , and *TRAP* parasite antigens. Collections took place at Ndangueng, Cameroon and Benguia, Gabon between November 2019 and July 2021, yielding infected *Anopheles gambiae* at both sites in addition to *Anopheles moucheti* in Cameroon and *Anopheles funestus* in Gabon. We found no significant difference in the mean number of parasite strains in infected sympatric mosquito species from each site. However, *Pfs47* exhibited a significant difference in the prevalence of an amino acid polymorphism (I248L) between *An. moucheti* and *An. gambiae* in Cameroon. Polymorphisms at two additional positions (N272Y/I and P369H) displayed significant linkage disequilibrium with I248L, and there were significant differences in the distribution of *Pfs47* haplotypes between sympatric mosquito species at both sites. These findings demonstrate divergent selection on *P. falciparum* mediated by sympatric vector species, suggesting that parasite genotype significantly impacts local vectorial capacity by modulating species-specific vector competence.

